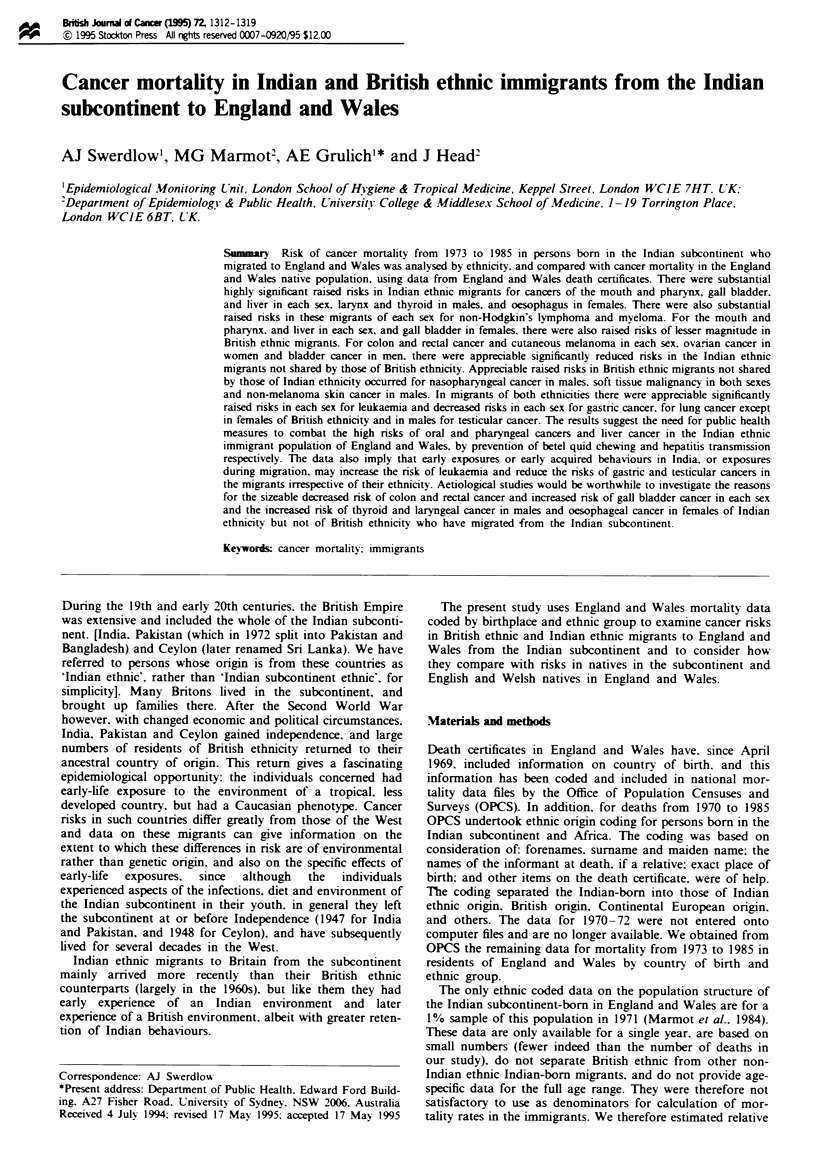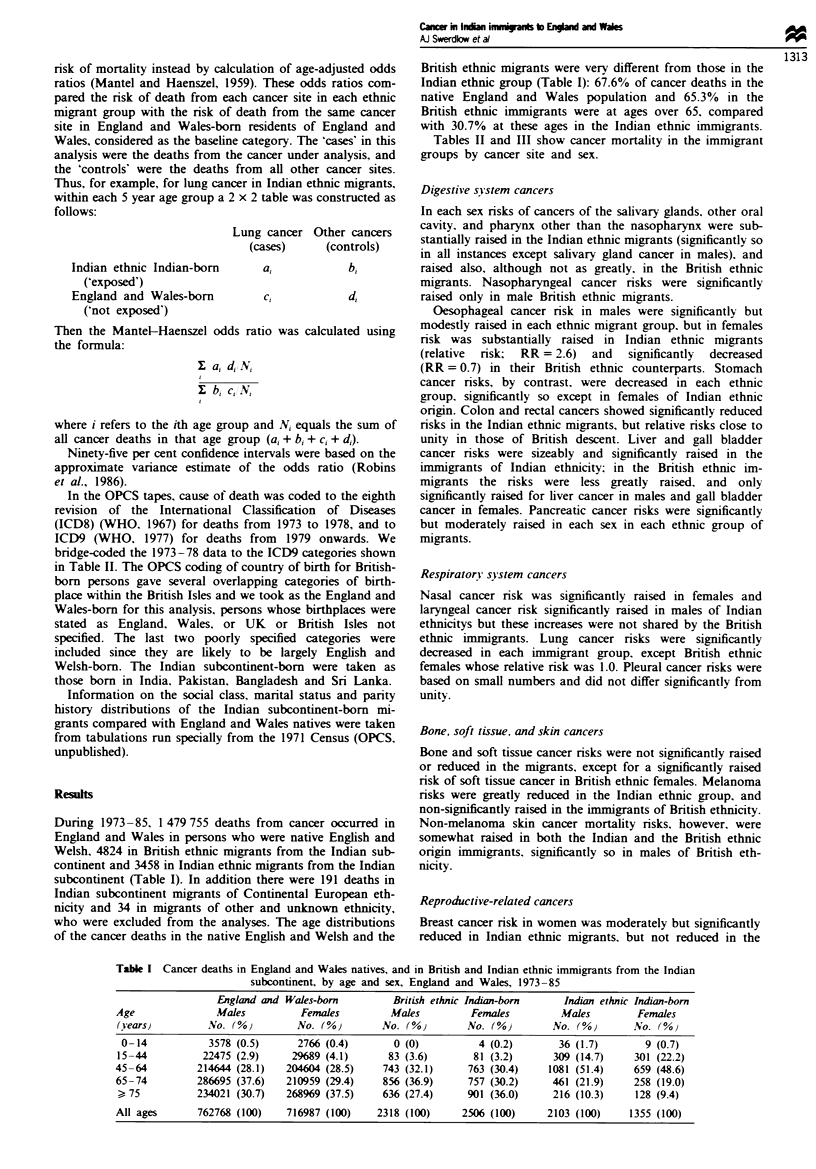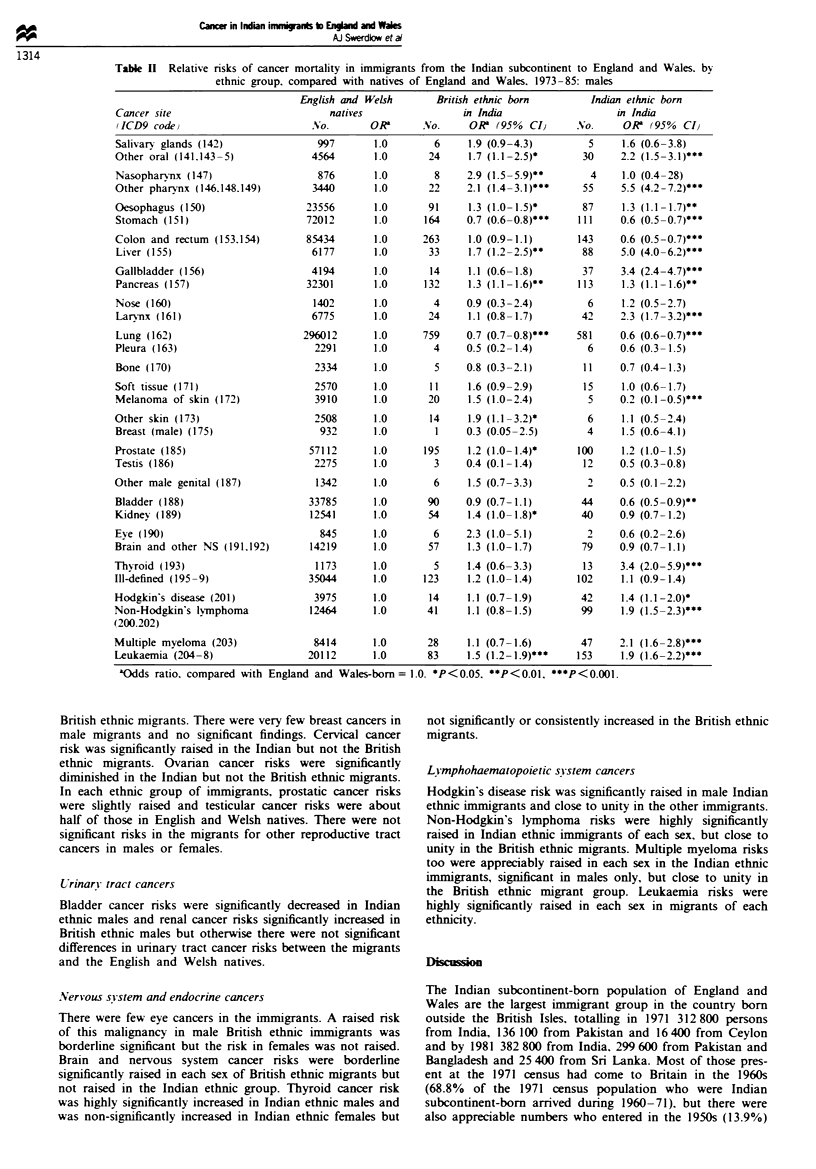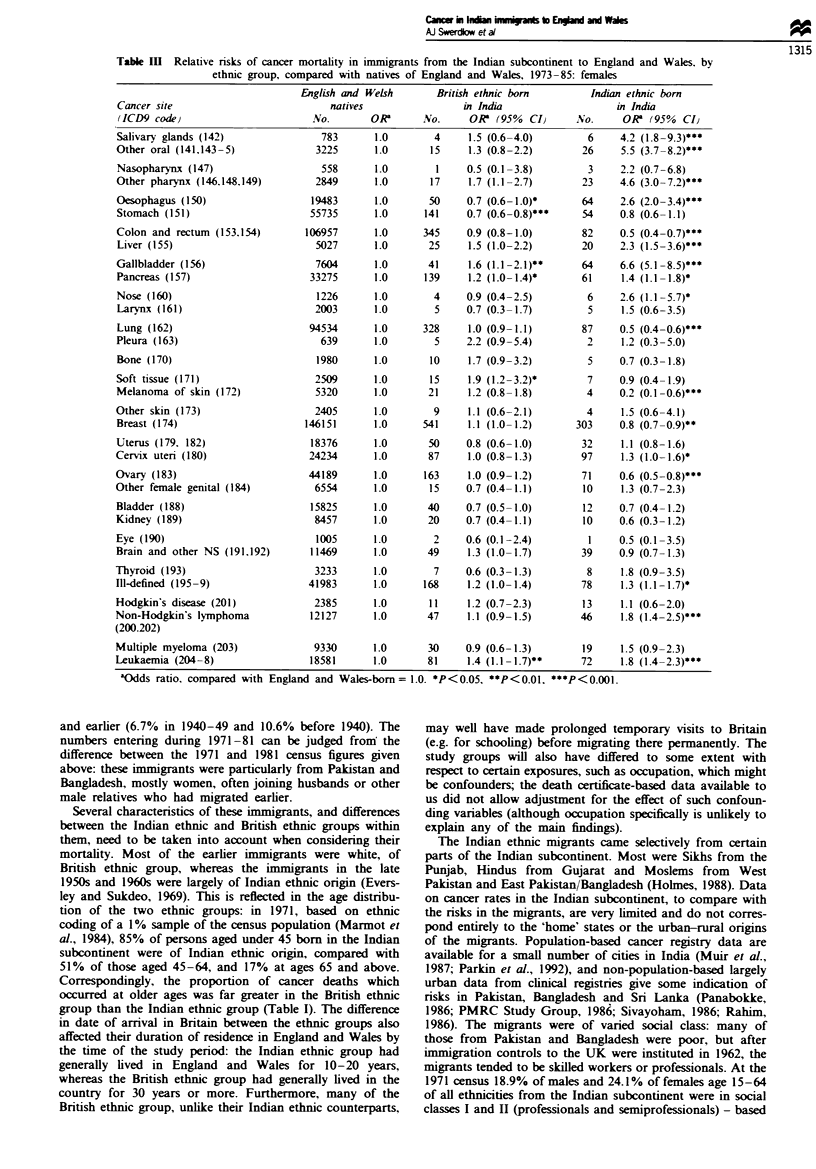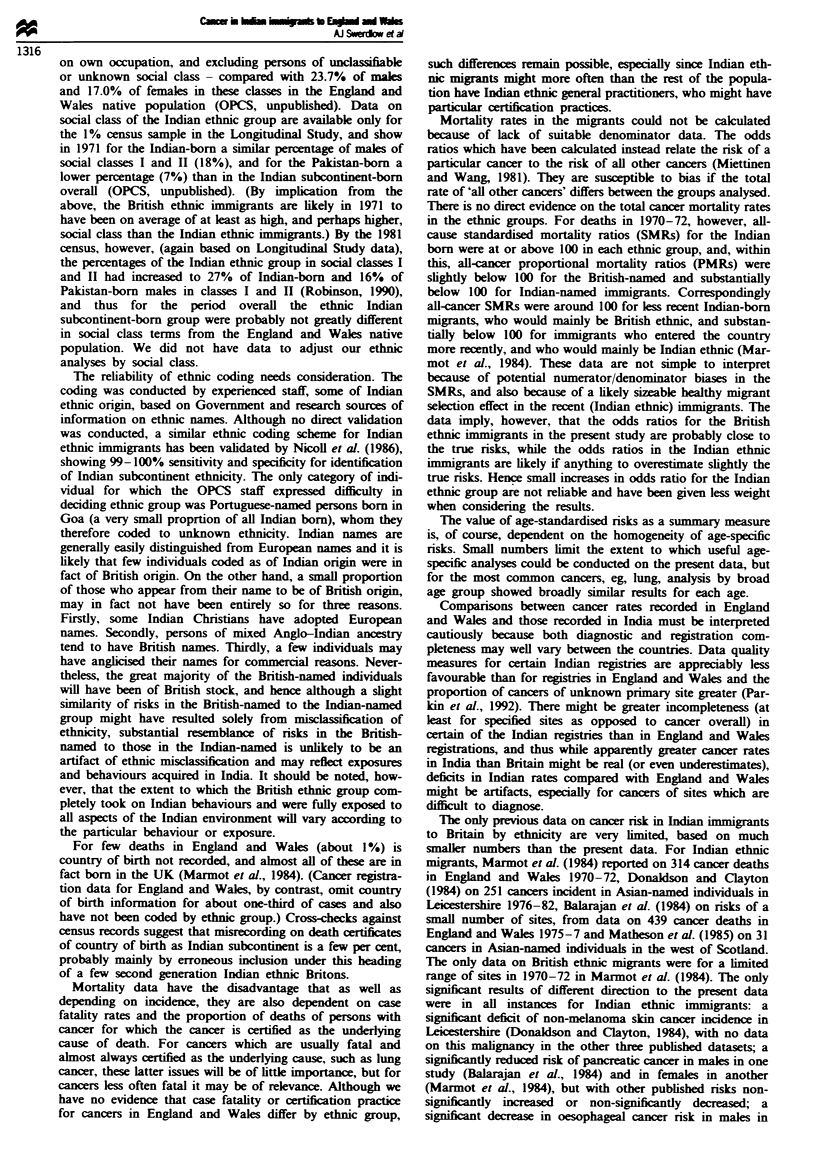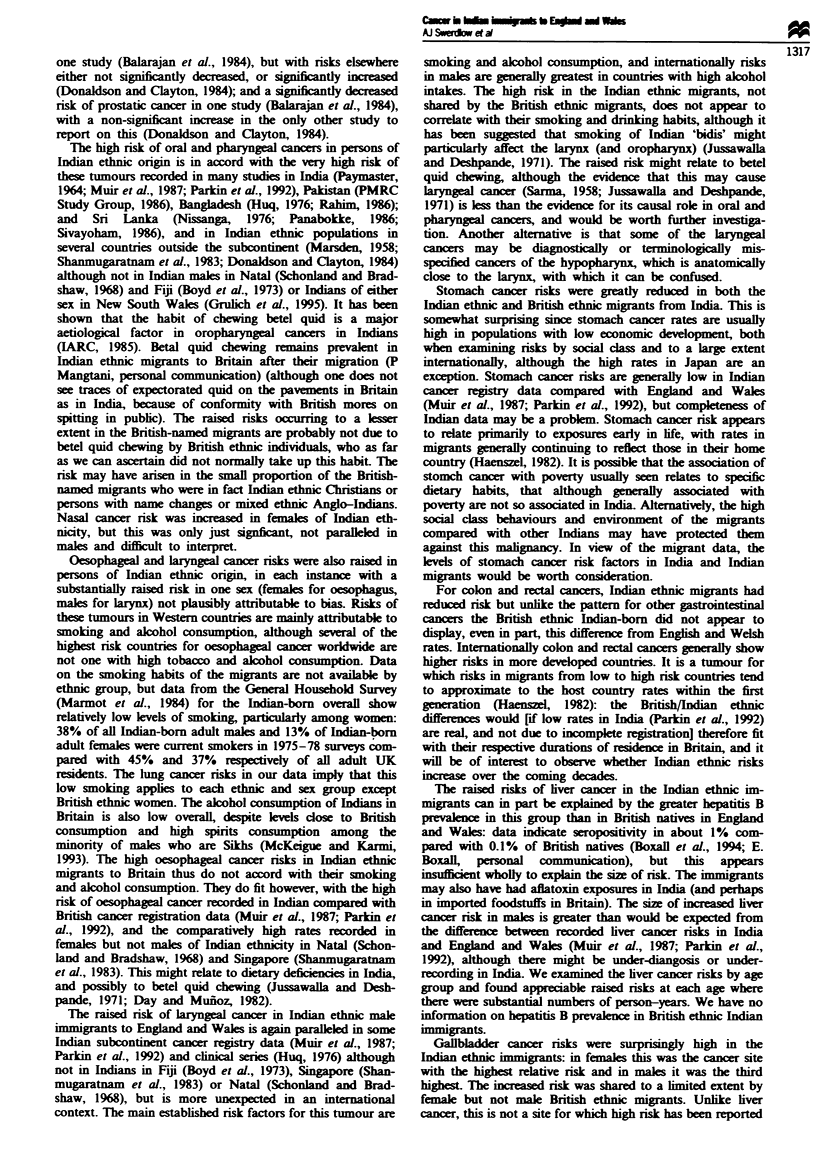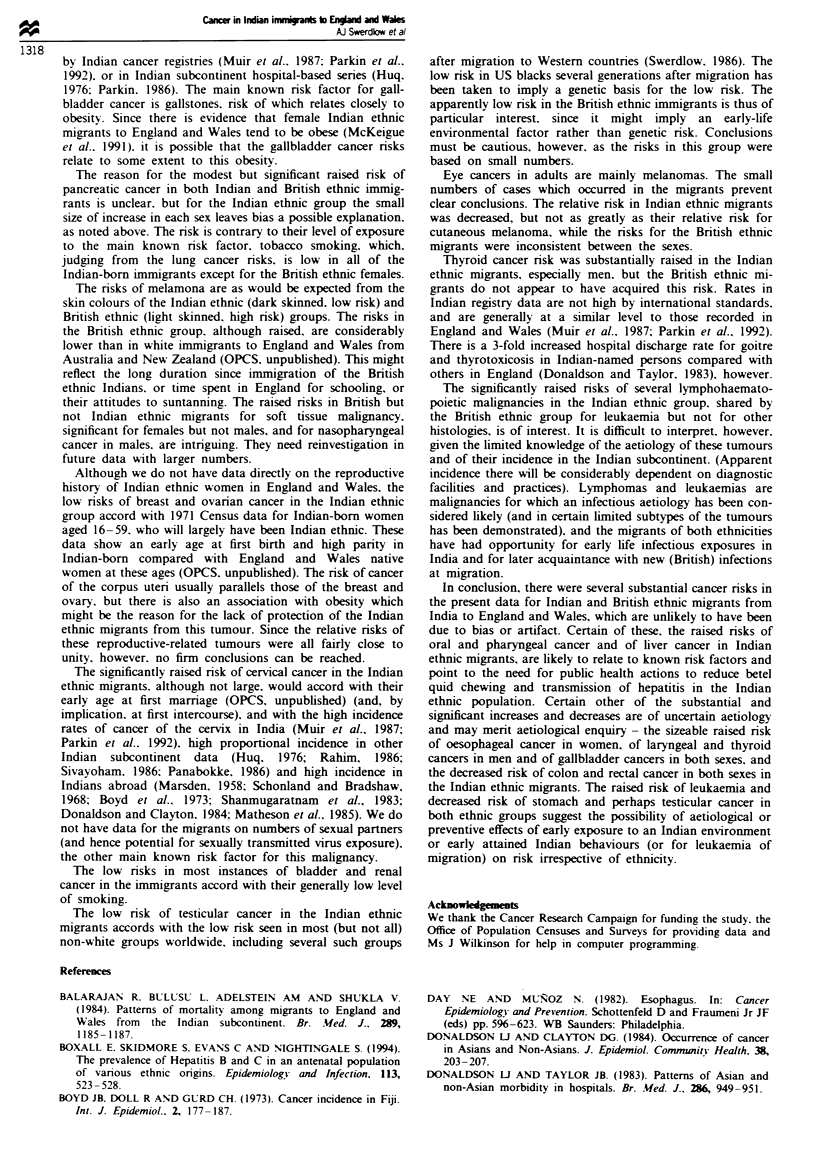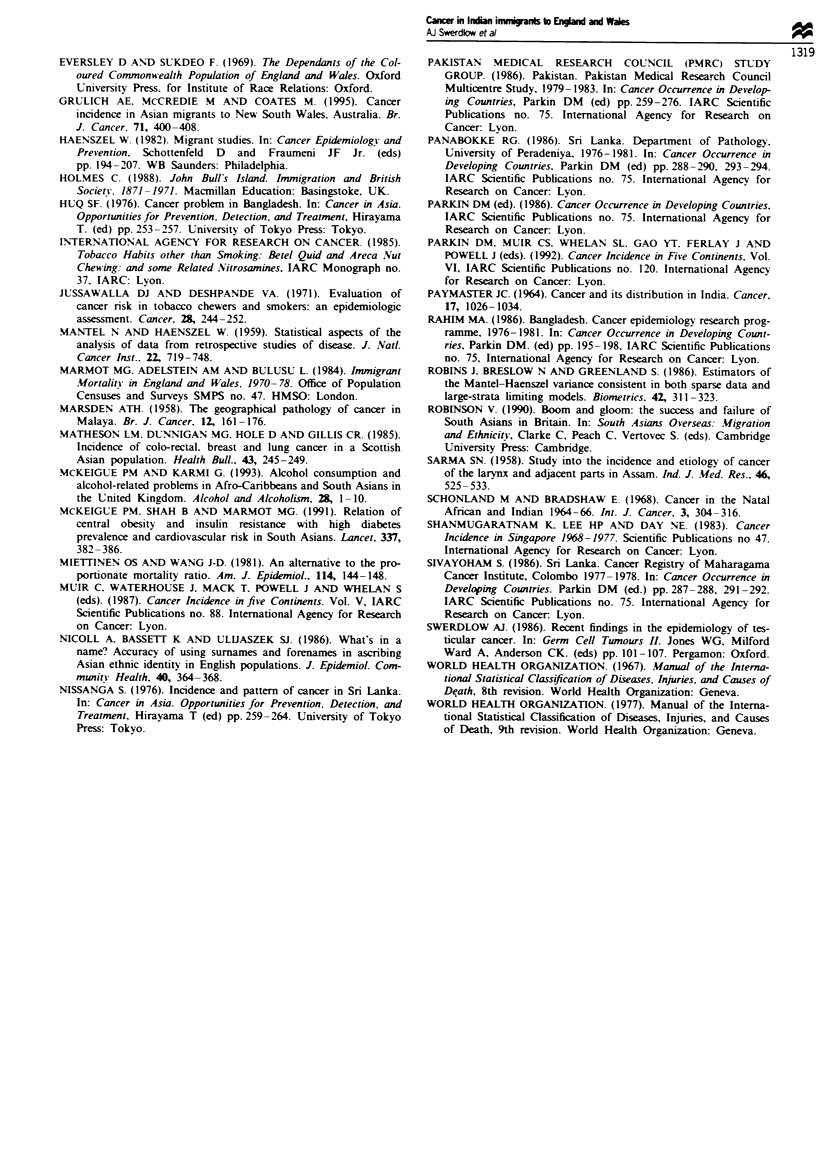# Cancer mortality in Indian and British ethnic immigrants from the Indian subcontinent to England and Wales.

**DOI:** 10.1038/bjc.1995.507

**Published:** 1995-11

**Authors:** A. J. Swerdlow, M. G. Marmot, A. E. Grulich, J. Head

**Affiliations:** Epidemiological Monitoring Unit, London School of Hygiene & Tropical Medicine, UK.

## Abstract

Risk of cancer mortality from 1973 to 1985 in persons born in the Indian subcontinent who migrated to England and Wales was analysed by ethnicity, and compared with cancer mortality in the England and Wales native population, using data from England and Wales death certificates. There were substantial highly significant raised risks in Indian ethnic migrants for cancers of the mouth and pharynx, gall bladder, and liver in each sex, larynx and thyroid in males, and oesophagus in females. There were also substantial raised risks in these migrants of each sex for non-Hodgkin's lymphoma and myeloma. For the mouth and pharynx, and liver in each sex, and gall bladder in females, there were also raised risks of lesser magnitude in British ethnic migrants. For colon and rectal cancer and cutaneous melanoma in each sex, ovarian cancer in women and bladder cancer in men, there were appreciable significantly reduced risks in the Indian ethnic migrants not shared by those of British ethnicity. Appreciable raised risks in British ethnic migrants not shared by those of Indian ethnicity occurred for nasopharyngeal cancer in males, soft tissue malignancy in both sexes and non-melanoma skin cancer in males. In migrants of both ethnicities there were appreciable significantly raised risks in each sex for leukaemia and decreased risks in each sex for gastric cancer, for lung cancer except in females of British ethnicity and in males for testicular cancer. The results suggest the need for public health measures to combat the high risks of oral and pharyngeal cancers and liver cancer in the Indian ethnic immigrant population of England and Wales, by prevention of betel quid chewing and hepatitis transmission respectively. The data also imply that early exposures or early acquired behaviours in India, or exposures during migration, may increase the risk of leukaemia and reduce the risks of gastric and testicular cancers in the migrants irrespective of their ethnicity. Aetiological studies would be worthwhile to investigate the reasons for the sizeable decreased risk of colon and rectal cancer and increased risk of gall bladder cancer in each sex and the increased risk of thyroid and laryngeal cancer in males and oesophageal cancer in females of Indian ethnicity but not of British ethnicity who have migrated from the Indian subcontinent.


British Journal of Cancer (1995) 72, 1312-1319
© 1995 Stockton Press All rights reserved 0007-0920/95 $12.00

### Cancer mortality in Indian and British ethnic immigrants from the Indian subcontinent to England and Wales

AJ Swerdlow[1], MG Marmot[2], AE Grulich[1]* and J Head[2]

[1]*Epidemiological Monitoring Unit, London School of Hygiene & Tropical Medicine, Keppel Street, London WC1E 7HT, UK;* [2]*Department of Epidemiology & Public Health, University College & Middlesex School of Medicine, 1–19 Torrington Place, London WC1E 6BT, UK.*

**Summary** Risk of cancer mortality from 1973 to 1985 in persons born in the Indian subcontinent who migrated to England and Wales was analysed by ethnicity, and compared with cancer mortality in the England and Wales native population, using data from England and Wales death certificates. There were substantial highly significant raised risks in Indian ethnic migrants for cancers of the mouth and pharynx, gall bladder, and liver in each sex, larynx and thyroid in males, and oesophagus in females. There were also substantial raised risks in these migrants of each sex for non-Hodgkin's lymphoma and myeloma. For the mouth and pharynx, and liver in each sex, and gall bladder in females, there were also raised risks of lesser magnitude in British ethnic migrants. For colon and rectal cancer and cutaneous melanoma in each sex, ovarian cancer in women and bladder cancer in men, there were appreciable significantly reduced risks in the Indian ethnic migrants not shared by those of British ethnicity. Appreciable raised risks in British ethnic migrants not shared by those of Indian ethnicity occurred for nasopharyngeal cancer in males, soft tissue malignancy in both sexes and non-melanoma skin cancer in males. In migrants of both ethnicities there were appreciable significantly raised risks in each sex for leukaemia and decreased risks in each sex for gastric cancer, for lung cancer except in females of British ethnicity and in males for testicular cancer. The results suggest the need for public health measures to combat the high risks of oral and pharyngeal cancers and liver cancer in the Indian ethnic immigrant population of England and Wales, by prevention of betel quid chewing and hepatitis transmission respectively. The data also imply that early exposures or early acquired behaviours in India, or exposures during migration, may increase the risk of leukaemia and reduce the risks of gastric and testicular cancers in the migrants irrespective of their ethnicity. Aetiological studies would be worthwhile to investigate the reasons for the sizeable decreased risk of colon and rectal cancer and increased risk of gall bladder cancer in each sex and the increased risk of thyroid and laryngeal cancer in males and oesophageal cancer in females of Indian ethnicity but not of British ethnicity who have migrated from the Indian subcontinent.

**Keywords:** cancer mortality; immigrants

During the 19th and early 20th centuries, the British Empire was extensive and included the whole of the Indian subcontinent. [India, Pakistan (which in 1972 split into Pakistan and Bangladesh) and Ceylon (later renamed Sri Lanka). We have referred to persons whose origin is from these countries as 'Indian ethnic', rather than 'Indian subcontinent ethnic', for simplicity]. Many Britons lived in the subcontinent, and brought up families there. After the Second World War however, with changed economic and political circumstances, India, Pakistan and Ceylon gained independence, and large numbers of residents of British ethnicity returned to their ancestral country of origin. This return gives a fascinating epidemiological opportunity: the individuals concerned had early-life exposure to the environment of a tropical, less developed country, but had a Caucasian phenotype. Cancer risks in such countries differ greatly from those of the West and data on these migrants can give information on the extent to which these differences in risk are of environmental rather than genetic origin, and also on the specific effects of early-life exposures, since although the individuals experienced aspects of the infections, diet and environment of the Indian subcontinent in their youth, in general they left the subcontinent at or before Independence (1947 for India and Pakistan, and 1948 for Ceylon), and have subsequently lived for several decades in the West.

Indian ethnic migrants to Britain from the subcontinent mainly arrived more recently than their British ethnic counterparts (largely in the 1960s), but like them they had early experience of an Indian environment and later experience of a British environment, albeit with greater retention of Indian behaviours.

Correspondence: AJ Swerdlow
*Present address: Department of Public Health, Edward Ford Building, A27 Fisher Road, University of Sydney, NSW 2006, Australia
Received 4 July 1994; revised 17 May 1995; accepted 17 May 1995

The present study uses England and Wales mortality data coded by birthplace and ethnic group to examine cancer risks in British ethnic and Indian ethnic migrants to England and Wales from the Indian subcontinent and to consider how they compare with risks in natives in the subcontinent and English and Welsh natives in England and Wales.

### Materials and methods

Death certificates in England and Wales have, since April 1969, included information on country of birth, and this information has been coded and included in national mortality data files by the Office of Population Censuses and Surveys (OPCS). In addition, for deaths from 1970 to 1985 OPCS undertook ethnic origin coding for persons born in the Indian subcontinent and Africa. The coding was based on consideration of: forenames, surname and maiden name; the names of the informant at death, if a relative; exact place of birth; and other items on the death certificate, were of help. The coding separated the Indian-born into those of Indian ethnic origin, British origin, Continental European origin, and others. The data for 1970–72 were not entered onto computer files and are no longer available. We obtained from OPCS the remaining data for mortality from 1973 to 1985 in residents of England and Wales by country of birth and ethnic group.

The only ethnic coded data on the population structure of the Indian subcontinent-born in England and Wales are for a 1% sample of this population in 1971 (Marmot *et al.*, 1984). These data are only available for a single year, are based on small numbers (fewer indeed than the number of deaths in our study), do not separate British ethnic from other non-Indian ethnic Indian-born migrants, and do not provide age-specific data for the full age range. They were therefore not satisfactory to use as denominators for calculation of mortality rates in the immigrants. We therefore estimated relative risk of mortality instead by calculation of age-adjusted odds ratios (Mantel and Haenszel, 1959). These odds ratios compared the risk of death from each cancer site in each ethnic migrant group with the risk of death from the same cancer site in England and Wales-born residents of England and Wales, considered as the baseline category. The 'cases' in this analysis were the deaths from the cancer under analysis, and the 'controls' were the deaths from all other cancer sites. Thus, for example, for lung cancer in Indian ethnic migrants, within each 5 year age group a 2 × 2 table was constructed as follows:

| | Lung cancer (cases) | Other cancers (controls) |
|---|---|---|
| Indian ethnic Indian-born ('exposed') | $a_i$ | $b_i$ |
| England and Wales-born ('not exposed') | $c_i$ | $d_i$ |

Then the Mantel-Haenszel odds ratio was calculated using the formula:

$$\frac{\sum_i a_i d_i / N_i}{\sum_i b_i c_i / N_i}$$

where $i$ refers to the $i$th age group and $N_i$ equals the sum of all cancer deaths in that age group $(a_i + b_i + c_i + d_i)$.

Ninety-five per cent confidence intervals were based on the approximate variance estimate of the odds ratio (Robins *et al.*, 1986).

In the OPCS tapes, cause of death was coded to the eighth revision of the International Classification of Diseases (ICD8) (WHO, 1967) for deaths from 1973 to 1978, and to ICD9 (WHO, 1977) for deaths from 1979 onwards. We bridge-coded the 1973–78 data to the ICD9 categories shown in Table II. The OPCS coding of country of birth for British-born persons gave several overlapping categories of birthplace within the British Isles and we took as the England and Wales-born for this analysis, persons whose birthplaces were stated as England, Wales, or UK or British Isles not specified. The last two poorly specified categories were included since they are likely to be largely English and Welsh-born. The Indian subcontinent-born were taken as those born in India, Pakistan, Bangladesh and Sri Lanka.

Information on the social class, marital status and parity history distributions of the Indian subcontinent-born migrants compared with England and Wales natives were taken from tabulations run specially from the 1971 Census (OPCS, unpublished).

### Results

During 1973–85, 1 479 755 deaths from cancer occurred in England and Wales in persons who were native English and Welsh, 4824 in British ethnic migrants from the Indian subcontinent and 3458 in Indian ethnic migrants from the Indian subcontinent (Table I). In addition there were 191 deaths in Indian subcontinent migrants of Continental European ethnicity and 34 in migrants of other and unknown ethnicity, who were excluded from the analyses. The age distributions of the cancer deaths in the native English and Welsh and the British ethnic migrants were very different from those in the Indian ethnic group (Table I): 67.6% of cancer deaths in the native England and Wales population and 65.3% in the British ethnic immigrants were at ages over 65, compared with 30.7% at these ages in the Indian ethnic immigrants.

Tables II and III show cancer mortality in the immigrant groups by cancer site and sex.

### *Digestive system cancers*

In each sex risks of cancers of the salivary glands, other oral cavity, and pharynx other than the nasopharynx were substantially raised in the Indian ethnic migrants (significantly so in all instances except salivary gland cancer in males), and raised also, although not as greatly, in the British ethnic migrants. Nasopharyngeal cancer risks were significantly raised only in male British ethnic migrants.

Oesophageal cancer risk in males were significantly but modestly raised in each ethnic migrant group, but in females risk was substantially raised in Indian ethnic migrants (relative risk; RR = 2.6) and significantly decreased (RR = 0.7) in their British ethnic counterparts. Stomach cancer risks, by contrast, were decreased in each ethnic group, significantly so except in females of Indian ethnic origin. Colon and rectal cancers showed significantly reduced risks in the Indian ethnic migrants, but relative risks close to unity in those of British descent. Liver and gall bladder cancer risks were sizeably and significantly raised in the immigrants of Indian ethnicity; in the British ethnic immigrants the risks were less greatly raised, and only significantly raised for liver cancer in males and gall bladder cancer in females. Pancreatic cancer risks were significantly but moderately raised in each sex in each ethnic group of migrants.

### *Respiratory system cancers*

Nasal cancer risk was significantly raised in females and laryngeal cancer risk significantly raised in males of Indian ethnicitys but these increases were not shared by the British ethnic immigrants. Lung cancer risks were significantly decreased in each immigrant group, except British ethnic females whose relative risk was 1.0. Pleural cancer risks were based on small numbers and did not differ significantly from unity.

### *Bone, soft tissue, and skin cancers*

Bone and soft tissue cancer risks were not significantly raised or reduced in the migrants, except for a significantly raised risk of soft tissue cancer in British ethnic females. Melanoma risks were greatly reduced in the Indian ethnic group, and non-significantly raised in the immigrants of British ethnicity. Non-melanoma skin cancer mortality risks, however, were somewhat raised in both the Indian and the British ethnic origin immigrants, significantly so in males of British ethnicity.

### *Reproductive-related cancers*

Breast cancer risk in women was moderately but significantly reduced in Indian ethnic migrants, but not reduced in the

**Table I** Cancer deaths in England and Wales natives, and in British and Indian ethnic immigrants from the Indian subcontinent, by age and sex, England and Wales, 1973–85

| *Age (years)* | *England and Wales-born Males No. (%)* | *England and Wales-born Females No. (%)* | *British ethnic Indian-born Males No. (%)* | *British ethnic Indian-born Females No. (%)* | *Indian ethnic Indian-born Males No. (%)* | *Indian ethnic Indian-born Females No. (%)* |
|---|---|---|---|---|---|---|
| 0–14 | 3578 (0.5) | 2766 (0.4) | 0 (0) | 4 (0.2) | 36 (1.7) | 9 (0.7) |
| 15–44 | 22475 (2.9) | 29689 (4.1) | 83 (3.6) | 81 (3.2) | 309 (14.7) | 301 (22.2) |
| 45–64 | 214644 (28.1) | 204604 (28.5) | 743 (32.1) | 763 (30.4) | 1081 (51.4) | 659 (48.6) |
| 65–74 | 286695 (37.6) | 210959 (29.4) | 856 (36.9) | 757 (30.2) | 461 (21.9) | 258 (19.0) |
| ⩾75 | 234021 (30.7) | 268969 (37.5) | 636 (27.4) | 901 (36.0) | 216 (10.3) | 128 (9.4) |
| All ages | 762768 (100) | 716987 (100) | 2318 (100) | 2506 (100) | 2103 (100) | 1355 (100) |

**Table II** Relative risks of cancer mortality in immigrants from the Indian subcontinent to England and Wales, by ethnic group, compared with natives of England and Wales, 1973–85: males

| Cancer site (ICD9 code) | English and Welsh natives No. | OR[a] | British ethnic born in India No. | OR[a] (95% CI) | Indian ethnic born in India No. | OR[a] (95% CI) |
|---|---|---|---|---|---|---|
| Salivary glands (142) | 997 | 1.0 | 6 | 1.9 (0.9–4.3) | 5 | 1.6 (0.6–3.8) |
| Other oral (141,143–5) | 4564 | 1.0 | 24 | 1.7 (1.1–2.5)* | 30 | 2.2 (1.5–3.1)*** |
| Nasopharynx (147) | 876 | 1.0 | 8 | 2.9 (1.5–5.9)** | 4 | 1.0 (0.4–28) |
| Other pharynx (146,148,149) | 3440 | 1.0 | 22 | 2.1 (1.4–3.1)*** | 55 | 5.5 (4.2–7.2)*** |
| Oesophagus (150) | 23556 | 1.0 | 91 | 1.3 (1.0–1.5)* | 87 | 1.3 (1.1–1.7)** |
| Stomach (151) | 72012 | 1.0 | 164 | 0.7 (0.6–0.8)*** | 111 | 0.6 (0.5–0.7)*** |
| Colon and rectum (153,154) | 85434 | 1.0 | 263 | 1.0 (0.9–1.1) | 143 | 0.6 (0.5–0.7)*** |
| Liver (155) | 6177 | 1.0 | 33 | 1.7 (1.2–2.5)** | 88 | 5.0 (4.0–6.2)*** |
| Gallbladder (156) | 4194 | 1.0 | 14 | 1.1 (0.6–1.8) | 37 | 3.4 (2.4–4.7)*** |
| Pancreas (157) | 32301 | 1.0 | 132 | 1.3 (1.1–1.6)** | 113 | 1.3 (1.1–1.6)** |
| Nose (160) | 1402 | 1.0 | 4 | 0.9 (0.3–2.4) | 6 | 1.2 (0.5–2.7) |
| Larynx (161) | 6775 | 1.0 | 24 | 1.1 (0.8–1.7) | 42 | 2.3 (1.7–3.2)*** |
| Lung (162) | 296012 | 1.0 | 759 | 0.7 (0.7–0.8)*** | 581 | 0.6 (0.6–0.7)*** |
| Pleura (163) | 2291 | 1.0 | 4 | 0.5 (0.2–1.4) | 6 | 0.6 (0.3–1.5) |
| Bone (170) | 2334 | 1.0 | 5 | 0.8 (0.3–2.1) | 11 | 0.7 (0.4–1.3) |
| Soft tissue (171) | 2570 | 1.0 | 11 | 1.6 (0.9–2.9) | 15 | 1.0 (0.6–1.7) |
| Melanoma of skin (172) | 3910 | 1.0 | 20 | 1.5 (1.0–2.4) | 5 | 0.2 (0.1–0.5)*** |
| Other skin (173) | 2508 | 1.0 | 14 | 1.9 (1.1–3.2)* | 6 | 1.1 (0.5–2.4) |
| Breast (male) (175) | 932 | 1.0 | 1 | 0.3 (0.05–2.5) | 4 | 1.5 (0.6–4.1) |
| Prostate (185) | 57112 | 1.0 | 195 | 1.2 (1.0–1.4)* | 100 | 1.2 (1.0–1.5) |
| Testis (186) | 2275 | 1.0 | 3 | 0.4 (0.1–1.4) | 12 | 0.5 (0.3–0.8) |
| Other male genital (187) | 1342 | 1.0 | 6 | 1.5 (0.7–3.3) | 2 | 0.5 (0.1–2.2) |
| Bladder (188) | 33785 | 1.0 | 90 | 0.9 (0.7–1.1) | 44 | 0.6 (0.5–0.9)** |
| Kidney (189) | 12541 | 1.0 | 54 | 1.4 (1.0–1.8)* | 40 | 0.9 (0.7–1.2) |
| Eye (190) | 845 | 1.0 | 6 | 2.3 (1.0–5.1) | 2 | 0.6 (0.2–2.6) |
| Brain and other NS (191,192) | 14219 | 1.0 | 57 | 1.3 (1.0–1.7) | 79 | 0.9 (0.7–1.1) |
| Thyroid (193) | 1173 | 1.0 | 5 | 1.4 (0.6–3.3) | 13 | 3.4 (2.0–5.9)*** |
| Ill-defined (195–9) | 35044 | 1.0 | 123 | 1.2 (1.0–1.4) | 102 | 1.1 (0.9–1.4) |
| Hodgkin's disease (201) | 3975 | 1.0 | 14 | 1.1 (0.7–1.9) | 42 | 1.4 (1.1–2.0)* |
| Non-Hodgkin's lymphoma (200,202) | 12464 | 1.0 | 41 | 1.1 (0.8–1.5) | 99 | 1.9 (1.5–2.3)*** |
| Multiple myeloma (203) | 8414 | 1.0 | 28 | 1.1 (0.7–1.6) | 47 | 2.1 (1.6–2.8)*** |
| Leukaemia (204–8) | 20112 | 1.0 | 83 | 1.5 (1.2–1.9)*** | 153 | 1.9 (1.6–2.2)*** |

[a]Odds ratio, compared with England and Wales-born = 1.0. *$P<0.05$, **$P<0.01$, ***$P<0.001$.

British ethnic migrants. There were very few breast cancers in male migrants and no significant findings. Cervical cancer risk was significantly raised in the Indian but not the British ethnic migrants. Ovarian cancer risks were significantly diminished in the Indian but not the British ethnic migrants. In each ethnic group of immigrants, prostatic cancer risks were slightly raised and testicular cancer risks were about half of those in English and Welsh natives. There were not significant risks in the migrants for other reproductive tract cancers in males or females.

*Urinary tract cancers*

Bladder cancer risks were significantly decreased in Indian ethnic males and renal cancer risks significantly increased in British ethnic males but otherwise there were not significant differences in urinary tract cancer risks between the migrants and the English and Welsh natives.

*Nervous system and endocrine cancers*

There were few eye cancers in the immigrants. A raised risk of this malignancy in male British ethnic immigrants was borderline significant but the risk in females was not raised. Brain and nervous system cancer risks were borderline significantly raised in each sex of British ethnic migrants but not raised in the Indian ethnic group. Thyroid cancer risk was highly significantly increased in Indian ethnic males and was non-significantly increased in Indian ethnic females but not significantly or consistently increased in the British ethnic migrants.

*Lymphohaematopoietic system cancers*

Hodgkin's disease risk was significantly raised in male Indian ethnic immigrants and close to unity in the other immigrants. Non-Hodgkin's lymphoma risks were highly significantly raised in Indian ethnic immigrants of each sex, but close to unity in the British ethnic migrants. Multiple myeloma risks too were appreciably raised in each sex in the Indian ethnic immigrants, significant in males only, but close to unity in the British ethnic migrant group. Leukaemia risks were highly significantly raised in each sex in migrants of each ethnicity.

### Discussion

The Indian subcontinent-born population of England and Wales are the largest immigrant group in the country born outside the British Isles, totalling in 1971 312 800 persons from India, 136 100 from Pakistan and 16 400 from Ceylon and by 1981 382 800 from India, 299 600 from Pakistan and Bangladesh and 25 400 from Sri Lanka. Most of those present at the 1971 census had come to Britain in the 1960s (68.8% of the 1971 census population who were Indian subcontinent-born arrived during 1960–71), but there were also appreciable numbers who entered in the 1950s (13.9%)

**Table III** Relative risks of cancer mortality in immigrants from the Indian subcontinent to England and Wales, by ethnic group, compared with natives of England and Wales, 1973–85: females

| *Cancer site (ICD9 code)* | *English and Welsh natives* No. | *OR*[a] | *British ethnic born in India* No. | *OR*[a] *(95% CI)* | *Indian ethnic born in India* No. | *OR*[a] *(95% CI)* |
|---|---|---|---|---|---|---|
| Salivary glands (142) | 783 | 1.0 | 4 | 1.5 (0.6–4.0) | 6 | 4.2 (1.8–9.3)*** |
| Other oral (141,143–5) | 3225 | 1.0 | 15 | 1.3 (0.8–2.2) | 26 | 5.5 (3.7–8.2)*** |
| Nasopharynx (147) | 558 | 1.0 | 1 | 0.5 (0.1–3.8) | 3 | 2.2 (0.7–6.8) |
| Other pharynx (146,148,149) | 2849 | 1.0 | 17 | 1.7 (1.1–2.7) | 23 | 4.6 (3.0–7.2)*** |
| Oesophagus (150) | 19483 | 1.0 | 50 | 0.7 (0.6–1.0)* | 64 | 2.6 (2.0–3.4)*** |
| Stomach (151) | 55735 | 1.0 | 141 | 0.7 (0.6–0.8)*** | 54 | 0.8 (0.6–1.1) |
| Colon and rectum (153,154) | 106957 | 1.0 | 345 | 0.9 (0.8–1.0) | 82 | 0.5 (0.4–0.7)*** |
| Liver (155) | 5027 | 1.0 | 25 | 1.5 (1.0–2.2) | 20 | 2.3 (1.5–3.6)*** |
| Gallbladder (156) | 7604 | 1.0 | 41 | 1.6 (1.1–2.1)** | 64 | 6.6 (5.1–8.5)*** |
| Pancreas (157) | 33275 | 1.0 | 139 | 1.2 (1.0–1.4)* | 61 | 1.4 (1.1–1.8)* |
| Nose (160) | 1226 | 1.0 | 4 | 0.9 (0.4–2.5) | 6 | 2.6 (1.1–5.7)* |
| Larynx (161) | 2003 | 1.0 | 5 | 0.7 (0.3–1.7) | 5 | 1.5 (0.6–3.5) |
| Lung (162) | 94534 | 1.0 | 328 | 1.0 (0.9–1.1) | 87 | 0.5 (0.4–0.6)*** |
| Pleura (163) | 639 | 1.0 | 5 | 2.2 (0.9–5.4) | 2 | 1.2 (0.3–5.0) |
| Bone (170) | 1980 | 1.0 | 10 | 1.7 (0.9–3.2) | 5 | 0.7 (0.3–1.8) |
| Soft tissue (171) | 2509 | 1.0 | 15 | 1.9 (1.2–3.2)* | 7 | 0.9 (0.4–1.9) |
| Melanoma of skin (172) | 5320 | 1.0 | 21 | 1.2 (0.8–1.8) | 4 | 0.2 (0.1–0.6)*** |
| Other skin (173) | 2405 | 1.0 | 9 | 1.1 (0.6–2.1) | 4 | 1.5 (0.6–4.1) |
| Breast (174) | 146151 | 1.0 | 541 | 1.1 (1.0–1.2) | 303 | 0.8 (0.7–0.9)** |
| Uterus (179, 182) | 18376 | 1.0 | 50 | 0.8 (0.6–1.0) | 32 | 1.1 (0.8–1.6) |
| Cervix uteri (180) | 24234 | 1.0 | 87 | 1.0 (0.8–1.3) | 97 | 1.3 (1.0–1.6)* |
| Ovary (183) | 44189 | 1.0 | 163 | 1.0 (0.9–1.2) | 71 | 0.6 (0.5–0.8)*** |
| Other female genital (184) | 6554 | 1.0 | 15 | 0.7 (0.4–1.1) | 10 | 1.3 (0.7–2.3) |
| Bladder (188) | 15825 | 1.0 | 40 | 0.7 (0.5–1.0) | 12 | 0.7 (0.4–1.2) |
| Kidney (189) | 8457 | 1.0 | 20 | 0.7 (0.4–1.1) | 10 | 0.6 (0.3–1.2) |
| Eye (190) | 1005 | 1.0 | 2 | 0.6 (0.1–2.4) | 1 | 0.5 (0.1–3.5) |
| Brain and other NS (191,192) | 11469 | 1.0 | 49 | 1.3 (1.0–1.7) | 39 | 0.9 (0.7–1.3) |
| Thyroid (193) | 3233 | 1.0 | 7 | 0.6 (0.3–1.3) | 8 | 1.8 (0.9–3.5) |
| Ill-defined (195–9) | 41983 | 1.0 | 168 | 1.2 (1.0–1.4) | 78 | 1.3 (1.1–1.7)* |
| Hodgkin's disease (201) | 2385 | 1.0 | 11 | 1.2 (0.7–2.3) | 13 | 1.1 (0.6–2.0) |
| Non-Hodgkin's lymphoma (200,202) | 12127 | 1.0 | 47 | 1.1 (0.9–1.5) | 46 | 1.8 (1.4–2.5)*** |
| Multiple myeloma (203) | 9330 | 1.0 | 30 | 0.9 (0.6–1.3) | 19 | 1.5 (0.9–2.3) |
| Leukaemia (204–8) | 18581 | 1.0 | 81 | 1.4 (1.1–1.7)** | 72 | 1.8 (1.4–2.3)*** |

[a]Odds ratio, compared with England and Wales-born = 1.0. *$P<0.05$, **$P<0.01$, ***$P<0.001$.

and earlier (6.7% in 1940–49 and 10.6% before 1940). The numbers entering during 1971–81 can be judged from the difference between the 1971 and 1981 census figures given above: these immigrants were particularly from Pakistan and Bangladesh, mostly women, often joining husbands or other male relatives who had migrated earlier.

Several characteristics of these immigrants, and differences between the Indian ethnic and British ethnic groups within them, need to be taken into account when considering their mortality. Most of the earlier immigrants were white, of British ethnic group, whereas the immigrants in the late 1950s and 1960s were largely of Indian ethnic origin (Eversley and Sukdeo, 1969). This is reflected in the age distribution of the two ethnic groups: in 1971, based on ethnic coding of a 1% sample of the census population (Marmot *et al*., 1984), 85% of persons aged under 45 born in the Indian subcontinent were of Indian ethnic origin, compared with 51% of those aged 45–64, and 17% at ages 65 and above. Correspondingly, the proportion of cancer deaths which occurred at older ages was far greater in the British ethnic group than the Indian ethnic group (Table I). The difference in date of arrival in Britain between the ethnic groups also affected their duration of residence in England and Wales by the time of the study period: the Indian ethnic group had generally lived in England and Wales for 10–20 years, whereas the British ethnic group had generally lived in the country for 30 years or more. Furthermore, many of the British ethnic group, unlike their Indian ethnic counterparts, may well have made prolonged temporary visits to Britain (e.g. for schooling) before migrating there permanently. The study groups will also have differed to some extent with respect to certain exposures, such as occupation, which might be confounders; the death certificate-based data available to us did not allow adjustment for the effect of such confounding variables (although occupation specifically is unlikely to explain any of the main findings).

The Indian ethnic migrants came selectively from certain parts of the Indian subcontinent. Most were Sikhs from the Punjab, Hindus from Gujarat and Moslems from West Pakistan and East Pakistan/Bangladesh (Holmes, 1988). Data on cancer rates in the Indian subcontinent, to compare with the risks in the migrants, are very limited and do not correspond entirely to the 'home' states or the urban–rural origins of the migrants. Population-based cancer registry data are available for a small number of cities in India (Muir *et al*., 1987; Parkin *et al*., 1992), and non-population-based largely urban data from clinical registries give some indication of risks in Pakistan, Bangladesh and Sri Lanka (Panabokke, 1986; PMRC Study Group, 1986; Sivayoham, 1986; Rahim, 1986). The migrants were of varied social class: many of those from Pakistan and Bangladesh were poor, but after immigration controls to the UK were instituted in 1962, the migrants tended to be skilled workers or professionals. At the 1971 census 18.9% of males and 24.1% of females age 15–64 of all ethnicities from the Indian subcontinent were in social classes I and II (professionals and semiprofessionals) – based on own occupation, and excluding persons of unclassifiable or unknown social class – compared with 23.7% of males and 17.0% of females in these classes in the England and Wales native population (OPCS, unpublished). Data on social class of the Indian ethnic group are available only for the 1% census sample in the Longitudinal Study, and show in 1971 for the Indian-born a similar percentage of males of social classes I and II (18%), and for the Pakistan-born a lower percentage (7%) than in the Indian subcontinent-born overall (OPCS, unpublished). (By implication from the above, the British ethnic immigrants are likely in 1971 to have been on average of at least as high, and perhaps higher, social class than the Indian ethnic immigrants.) By the 1981 census, however, (again based on Longitudinal Study data), the percentages of the Indian ethnic group in social classes I and II had increased to 27% of Indian-born and 16% of Pakistan-born males in classes I and II (Robinson, 1990), and thus for the period overall the ethnic Indian subcontinent-born group were probably not greatly different in social class terms from the England and Wales native population. We did not have data to adjust our ethnic analyses by social class.

The reliability of ethnic coding needs consideration. The coding was conducted by experienced staff, some of Indian ethnic origin, based on Government and research sources of information on ethnic names. Although no direct validation was conducted, a similar ethnic coding scheme for Indian ethnic immigrants has been validated by Nicoll *et al.* (1986), showing 99–100% sensitivity and specificity for identification of Indian subcontinent ethnicity. The only category of individual for which the OPCS staff expressed difficulty in deciding ethnic group was Portuguese-named persons born in Goa (a very small proprtion of all Indian born), whom they therefore coded to unknown ethnicity. Indian names are generally easily distinguished from European names and it is likely that few individuals coded as of Indian origin were in fact of British origin. On the other hand, a small proportion of those who appear from their name to be of British origin, may in fact not have been entirely so for three reasons. Firstly, some Indian Christians have adopted European names. Secondly, persons of mixed Anglo–Indian ancestry tend to have British names. Thirdly, a few individuals may have anglicised their names for commercial reasons. Nevertheless, the great majority of the British-named individuals will have been of British stock, and hence although a slight similarity of risks in the British-named to the Indian-named group might have resulted solely from misclassification of ethnicity, substantial resemblance of risks in the British-named to those in the Indian-named is unlikely to be an artifact of ethnic misclassification and may reflect exposures and behaviours acquired in India. It should be noted, however, that the extent to which the British ethnic group completely took on Indian behaviours and were fully exposed to all aspects of the Indian environment will vary according to the particular behaviour or exposure.

For few deaths in England and Wales (about 1%) is country of birth not recorded, and almost all of these are in fact born in the UK (Marmot *et al.*, 1984). (Cancer registration data for England and Wales, by contrast, omit country of birth information for about one-third of cases and also have not been coded by ethnic group.) Cross-checks against census records suggest that misrecording on death certificates of country of birth as Indian subcontinent is a few per cent, probably mainly by erroneous inclusion under this heading of a few second generation Indian ethnic Britons.

Mortality data have the disadvantage that as well as depending on incidence, they are also dependent on case fatality rates and the proportion of deaths of persons with cancer for which the cancer is certified as the underlying cause of death. For cancers which are usually fatal and almost always certified as the underlying cause, such as lung cancer, these latter issues will be of little importance, but for cancers less often fatal it may be of relevance. Although we have no evidence that case fatality or certification practice for cancers in England and Wales differ by ethnic group, such differences remain possible, especially since Indian ethnic migrants might more often than the rest of the population have Indian ethnic general practitioners, who might have particular certification practices.

Mortality rates in the migrants could not be calculated because of lack of suitable denominator data. The odds ratios which have been calculated instead relate the risk of a particular cancer to the risk of all other cancers (Miettinen and Wang, 1981). They are susceptible to bias if the total rate of 'all other cancers' differs between the groups analysed. There is no direct evidence on the total cancer mortality rates in the ethnic groups. For deaths in 1970–72, however, all-cause standardised mortality ratios (SMRs) for the Indian born were at or above 100 in each ethnic group, and, within this, all-cancer proportional mortality ratios (PMRs) were slightly below 100 for the British-named and substantially below 100 for Indian-named immigrants. Correspondingly all-cancer SMRs were around 100 for less recent Indian-born migrants, who would mainly be British ethnic, and substantially below 100 for immigrants who entered the country more recently, and who would mainly be Indian ethnic (Marmot *et al.*, 1984). These data are not simple to interpret because of potential numerator/denominator biases in the SMRs, and also because of a likely sizeable healthy migrant selection effect in the recent (Indian ethnic) immigrants. The data imply, however, that the odds ratios for the British ethnic immigrants in the present study are probably close to the true risks, while the odds ratios in the Indian ethnic immigrants are likely if anything to overestimate slightly the true risks. Hence small increases in odds ratio for the Indian ethnic group are not reliable and have been given less weight when considering the results.

The value of age-standardised risks as a summary measure is, of course, dependent on the homogeneity of age-specific risks. Small numbers limit the extent to which useful age-specific analyses could be conducted on the present data, but for the most common cancers, eg, lung, analysis by broad age group showed broadly similar results for each age.

Comparisons between cancer rates recorded in England and Wales and those recorded in India must be interpreted cautiously because both diagnostic and registration completeness may well vary between the countries. Data quality measures for certain Indian registries are appreciably less favourable than for registries in England and Wales and the proportion of cancers of unknown primary site greater (Parkin *et al.*, 1992). There might be greater incompleteness (at least for specified sites as opposed to cancer overall) in certain of the Indian registries than in England and Wales registrations, and thus while apparently greater cancer rates in India than Britain might be real (or even underestimates), deficits in Indian rates compared with England and Wales might be artifacts, especially for cancers of sites which are difficult to diagnose.

The only previous data on cancer risk in Indian immigrants to Britain by ethnicity are very limited, based on much smaller numbers than the present data. For Indian ethnic migrants, Marmot *et al.* (1984) reported on 314 cancer deaths in England and Wales 1970–72, Donaldson and Clayton (1984) on 251 cancers incident in Asian-named individuals in Leicestershire 1976–82, Balarajan *et al.* (1984) on risks of a small number of sites, from data on 439 cancer deaths in England and Wales 1975–7 and Matheson *et al.* (1985) on 31 cancers in Asian-named individuals in the west of Scotland. The only data on British ethnic migrants were for a limited range of sites in 1970–72 in Marmot *et al.* (1984). The only significant results of different direction to the present data were in all instances for Indian ethnic immigrants: a significant deficit of non-melanoma skin cancer incidence in Leicestershire (Donaldson and Clayton, 1984), with no data on this malignancy in the other three published datasets; a significantly reduced risk of pancreatic cancer in males in one study (Balarajan *et al.*, 1984) and in females in another (Marmot *et al.*, 1984), but with other published risks non-significantly increased or non-significantly decreased; a significant decrease in oesophageal cancer risk in males in one study (Balarajan *et al.*, 1984), but with risks elsewhere either not significantly decreased, or significantly increased (Donaldson and Clayton, 1984); and a significantly decreased risk of prostatic cancer in one study (Balarajan *et al.*, 1984), with a non-significant increase in the only other study to report on this (Donaldson and Clayton, 1984).

The high risk of oral and pharyngeal cancers in persons of Indian ethnic origin is in accord with the very high risk of these tumours recorded in many studies in India (Paymaster, 1964; Muir *et al.*, 1987; Parkin *et al.*, 1992), Pakistan (PMRC Study Group, 1986), Bangladesh (Huq, 1976; Rahim, 1986); and Sri Lanka (Nissanga, 1976; Panabokke, 1986; Sivayoham, 1986), and in Indian ethnic populations in several countries outside the subcontinent (Marsden, 1958; Shanmugaratnam *et al.*, 1983; Donaldson and Clayton, 1984) although not in Indian males in Natal (Schonland and Bradshaw, 1968) and Fiji (Boyd *et al.*, 1973) or Indians of either sex in New South Wales (Grulich *et al.*, 1995). It has been shown that the habit of chewing betel quid is a major aetiological factor in oropharyngeal cancers in Indians (IARC, 1985). Betal quid chewing remains prevalent in Indian ethnic migrants to Britain after their migration (P Mangtani, personal communication) (although one does not see traces of expectorated quid on the pavements in Britain as in India, because of conformity with British mores on spitting in public). The raised risks occurring to a lesser extent in the British-named migrants are probably not due to betel quid chewing by British ethnic individuals, who as far as we can ascertain did not normally take up this habit. The risk may have arisen in the small proportion of the British-named migrants who were in fact Indian ethnic Christians or persons with name changes or mixed ethnic Anglo-Indians. Nasal cancer risk was increased in females of Indian ethnicity, but this was only just signficant, not paralleled in males and difficult to interpret.

Oesophageal and laryngeal cancer risks were also raised in persons of Indian ethnic origin, in each instance with a substantially raised risk in one sex (females for oesophagus, males for larynx) not plausibly attributable to bias. Risks of these tumours in Western countries are mainly attributable to smoking and alcohol consumption, although several of the highest risk countries for oesophageal cancer worldwide are not one with high tobacco and alcohol consumption. Data on the smoking habits of the migrants are not available by ethnic group, but data from the General Household Survey (Marmot *et al.*, 1984) for the Indian-born overall show relatively low levels of smoking, particularly among women: 38% of all Indian-born adult males and 13% of Indian-born adult females were current smokers in 1975–78 surveys compared with 45% and 37% respectively of all adult UK residents. The lung cancer risks in our data imply that this low smoking applies to each ethnic and sex group except British ethnic women. The alcohol consumption of Indians in Britain is also low overall, despite levels close to British consumption and high spirits consumption among the minority of males who are Sikhs (McKeigue and Karmi, 1993). The high oesophageal cancer risks in Indian ethnic migrants to Britain thus do not accord with their smoking and alcohol consumption. They do fit however, with the high risk of oesophageal cancer recorded in Indian compared with British cancer registration data (Muir *et al.*, 1987; Parkin *et al.*, 1992), and the comparatively high rates recorded in females but not males of Indian ethnicity in Natal (Schonland and Bradshaw, 1968) and Singapore (Shanmugaratnam *et al.*, 1983). This might relate to dietary deficiencies in India, and possibly to betel quid chewing (Jussawalla and Deshpande, 1971; Day and Muñoz, 1982).

The raised risk of laryngeal cancer in Indian ethnic male immigrants to England and Wales is again paralleled in some Indian subcontinent cancer registry data (Muir *et al.*, 1987; Parkin *et al.*, 1992) and clinical series (Huq, 1976) although not in Indians in Fiji (Boyd *et al.*, 1973), Singapore (Shanmugaratnam *et al.*, 1983) or Natal (Schonland and Bradshaw, 1968), but is more unexpected in an international context. The main established risk factors for this tumour are smoking and alcohol consumption, and internationally risks in males are generally greatest in countries with high alcohol intakes. The high risk in the Indian ethnic migrants, not shared by the British ethnic migrants, does not appear to correlate with their smoking and drinking habits, although it has been suggested that smoking of Indian 'bidis' might particularly affect the larynx (and oropharynx) (Jussawalla and Deshpande, 1971). The raised risk might relate to betel quid chewing, although the evidence that this may cause laryngeal cancer (Sarma, 1958; Jussawalla and Deshpande, 1971) is less than the evidence for its causal role in oral and pharyngeal cancers, and would be worth further investigation. Another alternative is that some of the laryngeal cancers may be diagnostically or terminologically misspecified cancers of the hypopharynx, which is anatomically close to the larynx, with which it can be confused.

Stomach cancer risks were greatly reduced in both the Indian ethnic and British ethnic migrants from India. This is somewhat surprising since stomach cancer rates are usually high in populations with low economic development, both when examining risks by social class and to a large extent internationally, although the high rates in Japan are an exception. Stomach cancer risks are generally low in Indian cancer registry data compared with England and Wales (Muir *et al.*, 1987; Parkin *et al.*, 1992), but completeness of Indian data may be a problem. Stomach cancer risk appears to relate primarily to exposures early in life, with rates in migrants generally continuing to reflect those in their home country (Haenszel, 1982). It is possible that the association of stomch cancer with poverty usually seen relates to specific dietary habits, that although generally associated with poverty are not so associated in India. Alternatively, the high social class behaviours and environment of the migrants compared with other Indians may have protected them against this malignancy. In view of the migrant data, the levels of stomach cancer risk factors in India and Indian migrants would be worth consideration.

For colon and rectal cancers, Indian ethnic migrants had reduced risk but unlike the pattern for other gastrointestinal cancers the British ethnic Indian-born did not appear to display, even in part, this difference from English and Welsh rates. Internationally colon and rectal cancers generally show higher risks in more developed countries. It is a tumour for which risks in migrants from low to high risk countries tend to approximate to the host country rates within the first generation (Haenszel, 1982): the British/Indian ethnic differences would [if low rates in India (Parkin *et al.*, 1992) are real, and not due to incomplete registration] therefore fit with their respective durations of residence in Britain, and it will be of interest to observe whether Indian ethnic risks increase over the coming decades.

The raised risks of liver cancer in the Indian ethnic immigrants can in part be explained by the greater hepatitis B prevalence in this group than in British natives in England and Wales: data indicate seropositivity in about 1% compared with 0.1% of British natives (Boxall *et al.*, 1994; E. Boxall, personal communication), but this appears insufficient wholly to explain the size of risk. The immigrants may also have had aflatoxin exposures in India (and perhaps in imported foodstuffs in Britain). The size of increased liver cancer risk in males is greater than would be expected from the difference between recorded liver cancer risks in India and England and Wales (Muir *et al.*, 1987; Parkin *et al.*, 1992), although there might be under-diangosis or under-recording in India. We examined the liver cancer risks by age group and found appreciable raised risks at each age where there were substantial numbers of person–years. We have no information on hepatitis B prevalence in British ethnic Indian immigrants.

Gallbladder cancer risks were surprisingly high in the Indian ethnic immigrants: in females this was the cancer site with the highest relative risk and in males it was the third highest. The increased risk was shared to a limited extent by female but not male British ethnic migrants. Unlike liver cancer, this is not a site for which high risk has been reported by Indian cancer registries (Muir *et al.*, 1987; Parkin *et al.*, 1992), or in Indian subcontinent hospital-based series (Huq, 1976; Parkin, 1986). The main known risk factor for gallbladder cancer is gallstones, risk of which relates closely to obesity. Since there is evidence that female Indian ethnic migrants to England and Wales tend to be obese (McKeigue *et al.*, 1991), it is possible that the gallbladder cancer risks relate to some extent to this obesity.

The reason for the modest but significant raised risk of pancreatic cancer in both Indian and British ethnic immigrants is unclear, but for the Indian ethnic group the small size of increase in each sex leaves bias a possible explanation, as noted above. The risk is contrary to their level of exposure to the main known risk factor, tobacco smoking, which, judging from the lung cancer risks, is low in all of the Indian-born immigrants except for the British ethnic females.

The risks of melamona are as would be expected from the skin colours of the Indian ethnic (dark skinned, low risk) and British ethnic (light skinned, high risk) groups. The risks in the British ethnic group, although raised, are considerably lower than in white immigrants to England and Wales from Australia and New Zealand (OPCS, unpublished). This might reflect the long duration since immigration of the British ethnic Indians, or time spent in England for schooling, or their attitudes to suntanning. The raised risks in British but not Indian ethnic migrants for soft tissue malignancy, significant for females but not males, and for nasopharyngeal cancer in males, are intriguing. They need reinvestigation in future data with larger numbers.

Although we do not have data directly on the reproductive history of Indian ethnic women in England and Wales, the low risks of breast and ovarian cancer in the Indian ethnic group accord with 1971 Census data for Indian-born women aged 16–59, who will largely have been Indian ethnic. These data show an early age at first birth and high parity in Indian-born compared with England and Wales native women at these ages (OPCS, unpublished). The risk of cancer of the corpus uteri usually parallels those of the breast and ovary, but there is also an association with obesity which might be the reason for the lack of protection of the Indian ethnic migrants from this tumour. Since the relative risks of these reproductive-related tumours were all fairly close to unity, however, no firm conclusions can be reached.

The significantly raised risk of cervical cancer in the Indian ethnic migrants, although not large, would accord with their early age at first marriage (OPCS, unpublished) (and, by implication, at first intercourse), and with the high incidence rates of cancer of the cervix in India (Muir *et al.*, 1987; Parkin *et al.*, 1992), high proportional incidence in other Indian subcontinent data (Huq, 1976; Rahim, 1986; Sivayoham, 1986; Panabokke, 1986) and high incidence in Indians abroad (Marsden, 1958; Schonland and Bradshaw, 1968; Boyd *et al.*, 1973; Shanmugaratnam *et al.*, 1983; Donaldson and Clayton, 1984; Matheson *et al.*, 1985). We do not have data for the migrants on numbers of sexual partners (and hence potential for sexually transmitted virus exposure), the other main known risk factor for this malignancy.

The low risks in most instances of bladder and renal cancer in the immigrants accord with their generally low level of smoking.

The low risk of testicular cancer in the Indian ethnic migrants accords with the low risk seen in most (but not all) non-white groups worldwide, including several such groups after migration to Western countries (Swerdlow, 1986). The low risk in US blacks several generations after migration has been taken to imply a genetic basis for the low risk. The apparently low risk in the British ethnic immigrants is thus of particular interest, since it might imply an early-life environmental factor rather than genetic risk. Conclusions must be cautious, however, as the risks in this group were based on small numbers.

Eye cancers in adults are mainly melanomas. The small numbers of cases which occurred in the migrants prevent clear conclusions. The relative risk in Indian ethnic migrants was decreased, but not as greatly as their relative risk for cutaneous melanoma, while the risks for the British ethnic migrants were inconsistent between the sexes.

Thyroid cancer risk was substantially raised in the Indian ethnic migrants, especially men, but the British ethnic migrants do not appear to have acquired this risk. Rates in Indian registry data are not high by international standards, and are generally at a similar level to those recorded in England and Wales (Muir *et al.*, 1987; Parkin *et al.*, 1992). There is a 3-fold increased hospital discharge rate for goitre and thyrotoxicosis in Indian-named persons compared with others in England (Donaldson and Taylor, 1983), however.

The significantly raised risks of several lymphohaematopoietic malignancies in the Indian ethnic group, shared by the British ethnic group for leukaemia but not for other histologies, is of interest. It is difficult to interpret, however, given the limited knowledge of the aetiology of these tumours and of their incidence in the Indian subcontinent. (Apparent incidence there will be considerably dependent on diagnostic facilities and practices). Lymphomas and leukaemias are malignancies for which an infectious aetiology has been considered likely (and in certain limited subtypes of the tumours has been demonstrated), and the migrants of both ethnicities have had opportunity for early life infectious exposures in India and for later acquaintance with new (British) infections at migration.

In conclusion, there were several substantial cancer risks in the present data for Indian and British ethnic migrants from India to England and Wales, which are unlikely to have been due to bias or artifact. Certain of these, the raised risks of oral and pharyngeal cancer and of liver cancer in Indian ethnic migrants, are likely to relate to known risk factors and point to the need for public health actions to reduce betel quid chewing and transmission of hepatitis in the Indian ethnic population. Certain other of the substantial and significant increases and decreases are of uncertain aetiology and may merit aetiological enquiry – the sizeable raised risk of oesophageal cancer in women, of laryngeal and thyroid cancers in men and of gallbladder cancers in both sexes, and the decreased risk of colon and rectal cancer in both sexes in the Indian ethnic migrants. The raised risk of leukaemia and decreased risk of stomach and perhaps testicular cancer in both ethnic groups suggest the possibility of aetiological or preventive effects of early exposure to an Indian environment or early attained Indian behaviours (or for leukaemia of migration) on risk irrespective of ethnicity.

**Acknowledgements**
We thank the Cancer Research Campaign for funding the study, the Office of Population Censuses and Surveys for providing data and Ms J Wilkinson for help in computer programming.

**References**

BALARAJAN R, BULUSU L, ADELSTEIN AM AND SHUKLA V. (1984). Patterns of mortality among migrants to England and Wales from the Indian subcontinent. *Br. Med. J.*, **289**, 1185–1187.

BOXALL E, SKIDMORE S, EVANS C AND NIGHTINGALE S. (1994). The prevalence of Hepatitis B and C in an antenatal population of various ethnic origins. *Epidemiology and Infection*, **113**, 523–528.

BOYD JB, DOLL R AND GURD CH. (1973). Cancer incidence in Fiji. *Int. J. Epidemiol.*, **2**, 177–187.

DAY NE AND MUÑOZ N. (1982). Esophagus. In: *Cancer Epidemiology and Prevention*. Schottenfeld D and Fraumeni Jr JF (eds) pp. 596–623. WB Saunders: Philadelphia.

DONALDSON LJ AND CLAYTON DG. (1984). Occurrence of cancer in Asians and Non-Asians. *J. Epidemiol. Community Health*, **38**, 203–207.

DONALDSON LJ AND TAYLOR JB. (1983). Patterns of Asian and non-Asian morbidity in hospitals. *Br. Med. J.*, **286**, 949–951.

EVERSLEY D AND SUKDEO F. (1969). *The Dependants of the Coloured Commonwealth Population of England and Wales*. Oxford University Press, for Institute of Race Relations: Oxford.

GRULICH AE, MCCREDIE M AND COATES M. (1995). Cancer incidence in Asian migrants to New South Wales, Australia. *Br. J. Cancer*, **71**, 400-408.

HAENSZEL W. (1982). Migrant studies. In: *Cancer Epidemiology and Prevention*, Schottenfeld D and Fraumeni JF Jr. (eds) pp. 194-207. WB Saunders: Philadelphia.

HOLMES C. (1988). *John Bull's Island. Immigration and British Society, 1871-1971*. Macmillan Education: Basingstoke, UK.

HUQ SF. (1976). Cancer problem in Bangladesh. In: *Cancer in Asia. Opportunities for Prevention, Detection, and Treatment*, Hirayama T. (ed) pp. 253-257. University of Tokyo Press: Tokyo.

INTERNATIONAL AGENCY FOR RESEARCH ON CANCER. (1985). *Tobacco Habits other than Smoking: Betel Quid and Areca Nut Chewing: and some Related Nitrosamines*, IARC Monograph no. 37, IARC: Lyon.

JUSSAWALLA DJ AND DESHPANDE VA. (1971). Evaluation of cancer risk in tobacco chewers and smokers: an epidemiologic assessment. *Cancer*, **28**, 244-252.

MANTEL N AND HAENSZEL W. (1959). Statistical aspects of the analysis of data from retrospective studies of disease. *J. Natl. Cancer Inst.*, **22**, 719-748.

MARMOT MG, ADELSTEIN AM AND BULUSU L. (1984). *Immigrant Mortality in England and Wales, 1970-78*. Office of Population Censuses and Surveys SMPS no. 47. HMSO: London.

MARSDEN ATH. (1958). The geographical pathology of cancer in Malaya. *Br. J. Cancer*, **12**, 161-176.

MATHESON LM, DUNNIGAN MG, HOLE D AND GILLIS CR. (1985). Incidence of colo-rectal, breast and lung cancer in a Scottish Asian population. *Health Bull.*, **43**, 245-249.

MCKEIGUE PM AND KARMI G. (1993). Alcohol consumption and alcohol-related problems in Afro-Caribbeans and South Asians in the United Kingdom. *Alcohol and Alcoholism*, **28**, 1-10.

MCKEIGUE PM, SHAH B AND MARMOT MG. (1991). Relation of central obesity and insulin resistance with high diabetes prevalence and cardiovascular risk in South Asians. *Lancet*, **337**, 382-386.

MIETTINEN OS AND WANG J-D. (1981). An alternative to the proportionate mortality ratio. *Am. J. Epidemiol.*, **114**, 144-148.

MUIR C, WATERHOUSE J, MACK T, POWELL J AND WHELAN S (eds). (1987). *Cancer Incidence in five Continents*. Vol. V, IARC Scientific Publications no. 88. International Agency for Research on Cancer: Lyon.

NICOLL A, BASSETT K AND ULIJASZEK SJ. (1986). What's in a name? Accuracy of using surnames and forenames in ascribing Asian ethnic identity in English populations. *J. Epidemiol. Community Health*, **40**, 364-368.

NISSANGA S. (1976). Incidence and pattern of cancer in Sri Lanka. In: *Cancer in Asia. Opportunities for Prevention, Detection, and Treatment*, Hirayama T (ed) pp. 259-264. University of Tokyo Press: Tokyo.

PAKISTAN MEDICAL RESEARCH COUNCIL (PMRC) STUDY GROUP. (1986). Pakistan. Pakistan Medical Research Council Multicentre Study, 1979-1983. In: *Cancer Occurrence in Developing Countries*, Parkin DM (ed) pp. 259-276. IARC Scientific Publications no. 75. International Agency for Research on Cancer: Lyon.

PANABOKKE RG. (1986). Sri Lanka. Department of Pathology, University of Peradeniya, 1976-1981. In: *Cancer Occurrence in Developing Countries*, Parkin DM (ed) pp. 288-290, 293-294. IARC Scientific Publications no. 75. International Agency for Research on Cancer: Lyon.

PARKIN DM (ed). (1986). *Cancer Occurrence in Developing Countries*, IARC Scientific Publications no. 75. International Agency for Research on Cancer: Lyon.

PARKIN DM, MUIR CS, WHELAN SL, GAO YT, FERLAY J AND POWELL J (eds). (1992). *Cancer Incidence in Five Continents*, Vol. VI, IARC Scientific Publications no. 120. International Agency for Research on Cancer: Lyon.

PAYMASTER JC. (1964). Cancer and its distribution in India. *Cancer*, **17**, 1026-1034.

RAHIM MA. (1986). Bangladesh. Cancer epidemiology research programme, 1976-1981. In: *Cancer Occurrence in Developing Countries*, Parkin DM. (ed) pp. 195-198, IARC Scientific Publications no. 75, International Agency for Research on Cancer: Lyon.

ROBINS J, BRESLOW N AND GREENLAND S. (1986). Estimators of the Mantel-Haenszel variance consistent in both sparse data and large-strata limiting models. *Biometrics*, **42**, 311-323.

ROBINSON V. (1990). Boom and gloom: the success and failure of South Asians in Britain. In: *South Asians Overseas: Migration and Ethnicity*, Clarke C, Peach C, Vertovec S. (eds). Cambridge University Press: Cambridge.

SARMA SN. (1958). Study into the incidence and etiology of cancer of the larynx and adjacent parts in Assam. *Ind. J. Med. Res.*, **46**, 525-533.

SCHONLAND M AND BRADSHAW E. (1968). Cancer in the Natal African and Indian 1964-66. *Int. J. Cancer*, **3**, 304-316.

SHANMUGARATNAM K, LEE HP AND DAY NE. (1983). *Cancer Incidence in Singapore 1968-1977*. Scientific Publications no 47. International Agency for Research on Cancer: Lyon.

SIVAYOHAM S. (1986). Sri Lanka. Cancer Registry of Maharagama Cancer Institute, Colombo 1977-1978. In: *Cancer Occurrence in Developing Countries*. Parkin DM (ed.) pp. 287-288, 291-292. IARC Scientific Publications no. 75. International Agency for Research on Cancer: Lyon.

SWERDLOW AJ. (1986). Recent findings in the epidemiology of testicular cancer. In: *Germ Cell Tumours II*. Jones WG, Milford Ward A, Anderson CK. (eds) pp. 101-107. Pergamon: Oxford.

WORLD HEALTH ORGANIZATION. (1967). *Manual of the International Statistical Classification of Diseases, Injuries, and Causes of Death*, 8th revision. World Health Organization: Geneva.

WORLD HEALTH ORGANIZATION. (1977). Manual of the International Statistical Classification of Diseases, Injuries, and Causes of Death, 9th revision. World Health Organization: Geneva.